# Evaluation of Delafloxacin against a Burkholderia pseudomallei Efflux Mutant Panel

**DOI:** 10.1128/spectrum.00903-22

**Published:** 2022-08-16

**Authors:** Sandra P. McCurdy, Nawarat Somprasong, Herbert P. Schweizer

**Affiliations:** a Melinta Therapeutics, Morristown, New Jersey, USA; b The Pathogen and Microbiome Institute, Northern Arizona University, Flagstaff, Arizona, USA; Georgia Institute of Technology

**Keywords:** *Burkholderia*, biothreat, efflux pumps, delafloxacin, fluoroquinolone

## Abstract

*In vitro* activities of delafloxacin and ciprofloxacin were evaluated against Burkholderia pseudomallei mutants expressing or lacking defined resistance-nodulation-cell division (RND) efflux pumps using CLSI methodology at pHs of 5.8 and 7.2. Delafloxacin MIC values were as much as 8-fold lower at pH 5.8 than those at pH 7.2, while ciprofloxacin MICs increased as much as 8-fold. The data from this study suggest that compared to ciprofloxacin, delafloxacin may have improved efflux avoidance, notably at acidic pH. In contrast to ciprofloxacin, delafloxacin may thus retain its therapeutic potential, even in BpeEF-OprC efflux-pump-expressing B. pseudomallei strains that compromise the use of fluoroquinolones, such as ciprofloxacin.

**IMPORTANCE** Resistance-nodulation-cell division (RND) efflux pumps play a major role in intrinsic and acquired antibiotic resistance in Burkholderia pseudomallei, and these pumps are its only known multidrug resistance determinants. Fluoroquinolones have performed poorly in clinical settings and are currently not recommended for treatment of B. pseudomallei infections. While the reasons for the poor clinical performance of this pathogen remain unclear, efflux may be partially responsible since fluoroquinolones like ciprofloxacin are prone to efflux by RND pumps, notably BpeEF-OprC. *In vitro* efficacy testing using a panel of efflux-proficient and efflux-deficient strains allows identification of fluoroquinolones that compared to ciprofloxacin are less prone to efflux.

## OBSERVATION

Burkholderia pseudomallei is an aerobic, non-spore-forming, nonfermenting Gram-negative opportunistic pathogen (1). It is the causative agent of melioidosis, a serious disease that affects humans and other animal species and has the potential to be used as a biothreat pathogen (2, 3). Intrinsic or high-level acquired resistance within *Burkholderia* species is often attributable to synergy between reduced penetration through the outer membrane and efflux from the cell (4–6). The clinically significant efflux pumps in B. pseudomallei belong to the resistance-nodulation-cell division (RND) superfamily (7). Sequenced strains encode at least 10 RND efflux systems, but only three of these, AmrAB-OprA, BpeAB-OprB, and BpeEF-OprC have clinical significance (7). AmrAB-OprA is expressed in the vast majority of environmental and clinical B. pseudomallei isolates and is responsible for the organism’s intrinsic aminoglycoside and macrolide resistance (7). AmrR regulatory mutants can also extrude doxycycline (8). The BpeAB-OprB efflux pump is expressed at low levels in wild-type strains, such as B. pseudomallei 1026b, but can be upregulated in BpeR mutants (9). The BpeAB-OprB efflux pump effluxes chloramphenicol, fluoroquinolones, macrolides, and tetracyclines, but only at low levels (9). More recently, AmrAB-OprA and BpeAB-OprB have been implicated in increased meropenem resistance in clinical isolates as the result of AmrR and BpeR regulatory mutations (10). BpeEF-OprC is not expressed in wild-type strains but is upregulated in BpeT and BpeS regulatory mutants (11–13). The BpeEF-OprC efflux pump is known to be the major fluoroquinolone resistance mechanism in B. pseudomallei (14), but it also confers resistance to chloramphenicol and trimethoprim (11), and when hyperexpressed or in concert with *folM* mutations, resistance to sulfamethoxazole has been observed (11). To our knowledge, there is no evidence for induction of the operons for *bpeAB-oprB* and *bpeEF-oprC* in B. pseudomallei by fluoroquinolone pump substrates, such as ciprofloxacin (9, 11).

Delafloxacin differs from the other fluoroquinolones, such as ciprofloxacin, in the absence of a protonatable substituent, which confers to the molecule a weak acid character. This property further increases delafloxacin’s potency in acidic environments and may result in improved efficacy against B. pseudomallei, which can survive within host cell organelles, such as lysosomes, where the pH is acidic (15). In a postexposure prophylaxis murine model of melioidosis, delafloxacin showed significantly greater survival than ceftazidime, an agent considered the standard of care. In addition, complete splenic clearance of the organism was observed with delafloxacin, which was not observed with ceftazidime-treated animals (16).

To confirm delafloxacin activity against resistant B. pseudomallei isolates as well as provide further evidence of differentiation from the other fluoroquinolones, delafloxacin and ciprofloxacin were tested against a panel of B. pseudomallei RND efflux mutants using the standard CLSI MIC methodology with pH adjustments to pHs 5.8 and 7.2 (17). The control strain, Escherichia coli ATCC 25922, was tested in parallel with the mutant strains and the delafloxacin and ciprofloxacin MIC results were within CLSI quality control ranges (18). The efflux pump mutant strains used in this study were derived from B. pseudomallei 1026b (19), and their construction was described previously (9, 14).

As demonstrated in previously published studies (20), delafloxacin MIC values were as much as 8-fold lower at pH 5.8 than those at pH 7.2 (Table 1). At both pH values, delafloxacin was not effluxed by AmrAB-OprA, whereas ciprofloxacin seems to be weakly effluxed by this pump when wild-type 1026b and Bp340 MIC values were compared (Table 1). Under both conditions, delafloxacin was noticeably effluxed by BpeAB-OprB only when upregulated in a BpeR regulatory mutant, as demonstrated by 1026b and Bp340 MIC values compared to Bp58 MIC results. Compared to wild-type 1026b, BpeEF-OprC upregulation in the BpeT regulatory mutant Bp282 resulted in increased resistance to delafloxacin and ciprofloxacin at both pH values. Overall, MIC values for delafloxacin were much lower at pH 5.8 than at pH 7.2, the opposite of what was observed for ciprofloxacin. It should be noted that when BpeAB-OprB (Bp58) and BpeEF-OprC (Bp282) were overexpressed, the MICs of delafloxacin and ciprofloxacin were comparably increased at both pH values compared to those obtained with 1026b, even though their absolute MIC values were very different. In contrast, when these MICs (e.g., Bp282) are compared to those obtained with the AmrAB-OprA BpeAB-OprB BpeEF-OprC triple mutant, Bp320, the MICs of delafloxacin were much less decreased at both pHs 5.8 and 7.2 than the ciprofloxacin MICs. This finding can be interpreted either as delafloxacin being less prone to efflux than ciprofloxacin, or similar to what was described in the near neighbor Burkholderia thailandensis (5, 6), the triple mutations in Bp320 led to outer membrane permutations that impacted ciprofloxacin more than delafloxacin.

**TABLE 1:** *In vitro* susceptibility of B. pseudomallei strains expressing or lacking defined efflux pumps

Strain	Genotype	Efflux pump(s) expressed^*a*^	MIC (μg/mL)^*b*^			
			Ciprofloxacin		Delafloxacin	
			pH 5.8	pH 7.2	pH 5.8	pH 7.2
*B. pseudomallei*						
1026b	*amrA^+^B^+^-oprA^+^ bpeA^+^B^+^-oprB^+^ bpeE^+^F^+^-oprC^+^*	AmrAB-OprA→, BpeAB-OprB→	8	1	0.0625	0.25
Bp340	Δ(*amrRAB-oprA*)	BpeAB-OprB→	4	0.5	0.0625	0.25
Bp227	Δ(*bpeAB-oprB*)	AmrAB-OprA→	2	0.5	0.015	0.0625
Bp207	Δ(*amrRAB-oprA*) Δ(*bpeAB-oprB*)	None known	0.5	0.125	0.015	0.03125
Bp58	Δ*bpeR* Δ(*amrRAB-oprA*)	BpeAB-OprB↑	16	2	0.25	1
Bp282	Δ(*amrRAB-oprA*) Δ(*bpeAB-oprB*) *bpeT*	BpeEF-OprC↑	128	16	0.5	4
Bp320	Δ(*amrRAB-oprA*) Δ(*bpeAB-oprB*) Δ(*bpeEF-oprC*) *bpeT*	None known	0.03125	0.015	0.008	0.015

*E. coli* ATCC 25922			0.0625	0.008	0.004	0.015

a Arrows do not indicate pump expression under the experimental conditions of the current study, but rather indicate previously established endogenous levels of efflux pump expression (→) or constitutive expression due to regulatory mutations of *bpeR* in Bp58 and *bpeT* in Bp282 (↑).

b MIC testing for each strain was performed in biological triplicate on 3 separate days. The reported values represent the mode of the three values read for each isolate.

In conclusion, as evidenced by MICs obtained at pHs 5.8 and pH 7.2, the *in vitro* efficacy of delafloxacin is superior to that of ciprofloxacin. The data from this study suggests that compared to ciprofloxacin, delafloxacin may have improved efflux avoidance, notably at acidic pH. In contrast to ciprofloxacin, delafloxacin may thus retain its therapeutic potential, even in BpeEF-OprC-expressing B. pseudomallei strains that compromise use of other fluoroquinolones. Further evaluation of delafloxacin in animal models of melioidosis is warranted.
